# Regional Delivery of Chimeric Antigen Receptor (CAR) T-Cells for Cancer Therapy

**DOI:** 10.3390/cancers9070092

**Published:** 2017-07-18

**Authors:** Praveen Sridhar, Fabio Petrocca

**Affiliations:** Department of Surgery, Boston University, Boston, MA 02118, USA; fabio.petrocca@gmail.com

**Keywords:** Chimeric Antigen Receptor T-Cell delivery, CAR-T, regional CAR-T delivery, solid tumor immunotherapy

## Abstract

Chimeric Antigen Receptor (CAR) T-cells are T-cells with recombinant receptors targeted to tumor antigens. CAR-T cell therapy has emerged as a mode of immunotherapy and is now being extensively explored in hematologic cancer. In contrast, CAR-T cell use in solid tumors has been hampered by multiple obstacles. Several approaches have been taken to circumvent these obstacles, including the regional delivery of CAR-T cells. Regional CAR-T cell delivery can theoretically compensate for poor T-cell trafficking and tumor antigen specificity while avoiding systemic toxicity associated with intravenous delivery. We reviewed completed clinical trials for the treatment of glioblastoma and metastatic colorectal cancer and examined the data in these studies for safety, efficacy, and potential advantages that regional delivery may confer over systemic delivery. Our appraisal of the available literature revealed that regional delivery of CAR-T cells in both glioblastoma and hepatic colorectal metastases was generally well tolerated and efficacious in select instances. We propose that the regional delivery of CAR-T cells is an area of potential growth in the solid tumor immunotherapy, and look towards future clinical trials in head and neck cancer, mesothelioma, and peritoneal carcinomatosis as the use of this technique expands.

## 1. Introduction

Chimeric Antigen Receptor (CAR) T-cells are T-cells with recombinant receptors that are the product of fused tumor antigen specific antibodies to the single chain fragment variable domains of the original T-cell receptor.

Modified T-cell therapy, including CAR-T cell therapy, has already emerged in the realm of hematologic cancers. The use of CAR-T cells in solid tumors has been slowed by several unique barriers including a lack of antigen specificity, inefficient T-cell trafficking, hostile tumor stromal environment, inhibitory cytokines and regulatory T-cells, and negative intrinsic regulators of the T-cell response [1]. While antigens for CAR-T cells in hematologic malignancies, most notably CD19 in B-cell malignancies and B-cell maturation antigen (BCMA) in multiple myeloma, are selectively expressed on target cancer cells and their cells of origin, targetable antigens in solid tumors are more elusive.

CARs currently being produced to target solid tumors are becoming increasingly specific, yet there is still concern for off-target effects. Advances made to second and third generation CAR-T cells have included transduction of additional costimulatory receptors, chimeric costimulatory receptors, inhibitory CARs, CARs specific to post-translational modifications, and transiently expressed CARs. These innovations, while promising, have only been examined in a pre-clinical setting [2,3,4,5,6,7,8]. The concept of transient CAR expression and inhibitory stimulation to overcome systemic toxicity will require intensive study on the affinity and titration of these antagonistic CARs. Furthermore, they give rise to the question of the durability of response that CAR-T cells may exhibit given unknown levels of persistence in the presence of negative regulators [9].

A lack of exclusive tumor antigen specificity is not the only characteristic of solid tumor-directed CARs that can lead to unwanted toxicity. Trafficking of CAR-T cells involves homing to and subsequently infiltrating tumor tissue. This process presents a significant barrier to the advancement of CAR-T therapies in solid tumors. Effective trafficking is dependent upon compatibility between tumor chemokines and chemokine receptors present on the CAR. Chemokine-receptor mismatch, as well as low levels of tumor chemokine secretion can lead to ineffective targeting of CAR-T cells to their target tissue [2,9]. Inefficient infiltration into target tissues relative to normal tissue after systemic administration of CAR-T cells raises concern for off-target, dose limiting toxicity [10].

To circumvent the difficulties presented by inefficient trafficking and systemic toxicity, several investigators have explored regional delivery of CAR-T cells directly to tumor tissue [11,12,13,14,15,16,17,18,19]. The concept of targeted delivery of chemotherapeutics to limit systemic toxicity has been shown to be effective in multiple previous clinical studies across several organ systems [20,21,22]. The regional delivery of CAR-T cells has emerged on the frontier of immunotherapy for solid tumors. We will review the current state of regional CAR-T cell delivery in the clinical setting.

## 2. Regional Delivery of CAR-T Cells in Clinical Practice

There is a paucity of completed clinical trials to date examining the role of regional CAR-T cell delivery to tumor tissues in human patients [12,13,17,19]. These trials have explored regional delivery within the central nervous system for glioblastoma and intrahepatically for liver metastases from primary colorectal cancer.

### 2.1. Gliobastoma

Glioblastoma is the most common brain tumor, and is generally an aggressive tumor with a median survival of only 15 months [15]. There has been an increasing interest in developing CAR modified cells for glioblastomas considering their refractory nature to chemotherapy, radiation therapy, and surgery. Antigen targets of interest have included interleukin-13 receptor alpha 2 (IL13Rα2) and EGFR on the basis that they are upregulated in more than 50% of glioblastoma multiforme [12,13,15]. While multiple authors have detailed the efficacy and safety of intracerebral delivery of EGFR variant III (EGFRvIII) targeting CAR-T cells and CAR-NK cells in a preclinical setting, there are only three clinical trials in which CAR-T cells have been delivered either intracranially or intrathecally for glioblastoma [12,14,15,19]. In all three clinical trials, investigators utilized zetakines, or mutated cytokines that bind target receptors, rather than antibodies with modified target domains [23]. In these trials, the authors used the Interleukin-13 (IL-13) cytokine containing a mutation that conferred a high affinity to the IL-13 receptor 2-alpha.

In the initial experience of CAR-T use in glioblastoma, Yaghoubi et al. treated a 57 year-old man with a grade IV glioblastoma multiforme [19]. This patient underwent resection of his primary tumor with diagnosis of recurrence via Magnetic Resonance Imaging (MRI) nine months following primary resection. Following his initial resection, the patient underwent leukapharesis and CD8 positive cytolytic T-cells were isolated and transduced with IL-13 zetakine receptor encoding plasmid DNA. At resection of the recurrent tumor, a Rickham reservoir catheter was inserted into the resection cavity. The patient then underwent catheter infusion of CAR-T cells for three days per week over a course of five weeks. The patient received a starting dose of 10^7^ cells and was gradually increased to a dose of 10^9^ cells as tolerated. He did experience progression of disease during therapy, at which point he received focal radiation and chemotherapy along with additional intralesional CAR-T doses. Subsequent tumor regression was seen on imaging 14 weeks later. There were no adverse events observed in this study during T-cell administration. CAR-T cell persistence was measured five weeks from the start of T-cell infusions using functional Positron Emission Tomography (PET) imaging that probed for a co-transfected reporter gene. Imaging revealed evidence of CAR-T cell persistence at the site of intralesional infusion as well as at sites of progression, suggesting that the CAR-T cells displayed trafficking ability after local infusion.

Brown et al. then performed a phase I trial in which regionally delivered IL13Rα2 targeted CAR-T cells were used to treat patients with recurrent glioblastoma [12]. This trial included three patients who had primary resection of glioblastoma multiforme tumors followed by recurrent disease. At the time of recurrence, these patients were treated with intracranial delivery of CAR-T cells into the resection cavity via Rickham catheter. In this protocol, 10^7^, 5 × 10^7^, and 10^8^ cells in a volume of 2 mL were injected over 5–10 min on three, non-consecutive days for the first week, followed by a dose of 10^8^ for nine additional doses over the course of the next four weeks. While primary outcomes evaluated were safety, feasibility, and toxicity, radiographic tumor volumes were also measured by MRI and combined Positron Emission Tomography and Computed Tomography (PET-CT). IL13Rα2 expression in tumor regions was quantified by immunohistochemistry (IHC), polymerase chain reaction (PCR), and flow-cytometry.

While there were no adverse events reported after the administration of 10^7^ and 5 × 10^7^ cells, one patient did suffer a headache after receiving 10^8^ cells. Another patient experienced reversible Grade 3 neurological events including changes to gait and tongue deviation with the same dose on the day after the final dose was administered. This complication required hospitalization and steroid treatment. It was unclear whether these events were solely secondary to T-cell therapy, as this patient did have progression of disease. This patient required a repeat craniotomy, at which point additional tumor was analyzed using IHC, PCR, and flow-cytometry. This analysis revealed that IL13Rα2 expression decreased significantly compared to pre-treatment levels, therefore recurrence of this patient’s disease may have been secondary to a non-IL13Rα2 cell subtype. Post treatment imaging conducted for detection of recurrence at the resection margins revealed that tumor did not recur in two of three patients. Unfortunately, this study did not assess for the duration of persistence of CAR-T cells within the target tissue and at other sites in the body.

**Figure 1:**
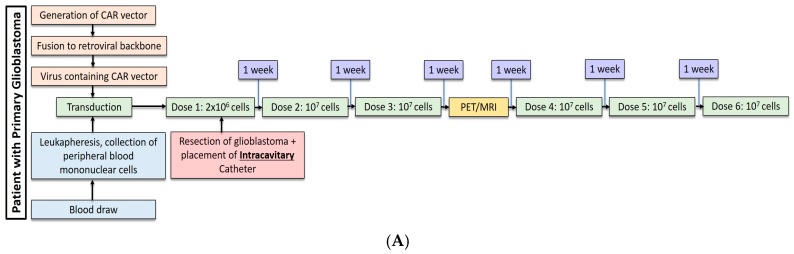

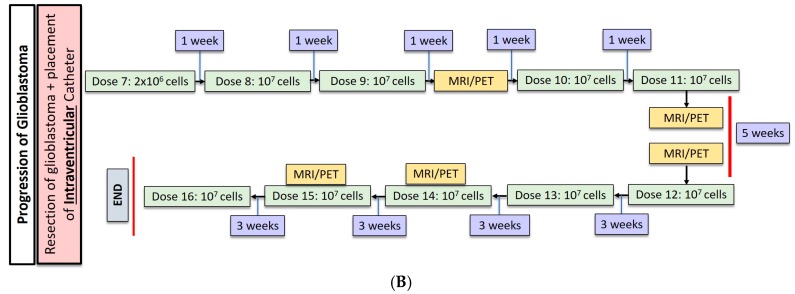
(**A**) Dose regimen for post-resection, intracavitary CAR-T cell delivery for glioblastoma. (**B**) Following progression of glioblastoma, an intraventricular catheter was placed for administration of 10 additional doses of CAR-T cells.

Most recently, Brown et al. conducted a second study in one patient with recurrent, multifocal glioblastoma who received IL13Rα2-targeted CAR-T cells via two different regional delivery routes [13]. In this trial, the IL13Rα2-targeted CAR-T cells included a costimulatory CD137 ligand as well as a modification of the Fc region to reduce off-target Fc region binding. The patient is a 50-year-old man with glioblastoma who had undergone resection, radiation, and temozolomide with disease recurrence six months after this therapy shown by imaging. At the onset of CAR-T therapy, the patient had five intracranial lesions, of which three were resected, including the largest lesion. CAR-T cells were then administered in a dose escalating fashion to a maximum dose of 10^7^ cells over six total infusions into a single tumor cavity (Figure 1A). Following approximately eight weeks of treatment, there was no radiographical evidence of tumor recurrence at the margins of the infused cavity. Two new lesions, however, recurred within each of the other two resection cavities. Additionally, the remaining two unresected lesions showed progression, and new spinal lesions developed. At this point, a second catheter was placed into the right lateral ventricle to improve CAR-T cell trafficking to these recurring lesions, and the patient received 10 additional intraventricular infusions over the course of 25 weeks (Figure 1B). In total, the duration of treatment from the administration of the first dose to the completion of the final dose was approximately 33 weeks. Following the initial three intraventricular infusions, all the intracranial and spinal masses reduced in volume. After five intraventricular infusions, all tumors decreased at least 77%. After completion of all 10 infusions, the original and recurrent tumors were measurable neither by MRI nor PET. This response was seen over the course of 7.5 months during active therapy; however; the patient was diagnosed with new metastases at four distinct sites that were nonadjacent to the original and recurrent tumors, as seen on imaging following the final intraventricular infusion. Importantly, CAR-T cell persistence was measured in the cerebrospinal fluid (CSF) immediately after each intraventricular infusion. The concentration of CAR-T cells increased by an average of ~7 cells/mm^3^ after each infusion. Following the first five intraventricular infusions, CAR-T cells were found to persist for at least seven days but had peaked two days following infusion. Levels of inflammatory cytokines (interferon-γ, tumor necrosis factor α, IL-2, IL-5, IL-6, IL-8, IL-10, chemokine ligand (CXCL) 9, CXCL10, CCR2, IL-1Rα) were also measured following infusions, and were found to be 10-fold higher compared to pre-infusion baseline levels. Though CAR-T cells did not show robust persistence within the CSF, the stimulation of the endogenous anti-tumor immune response by chemokines is hypothesized to have played a role in the regression of this patient’s tumor.

Throughout therapy, there were no reported toxic effects that were grade 3 or higher. The patient did experience nonspecific fatigue, myalgias, and olfactory auras that would be considered grade 1 or 2 adverse events if they were attributed to therapy.

### 2.2. Hepatic Metastases of Colorectal Adenocarcinoma

Greater than 50% of patients with colorectal cancer will develop liver metastases, and 25% of patients with colorectal cancer present with synchronous hepatic lesions. Surgical therapy can improve outcomes for patients with hepatic metastases but patients must meet multiple criteria for resection based on extent of disease and liver remnant volume [24]. This leaves many patients with conventional cytotoxic chemotherapy as their only safe option for treatment. It is hypothesized that the immunosuppressive qualities of the liver foster the development of hepatic metastases, and previous studies have shown that strong host T-cell responses are associated with improved survival in primary hepatic tumors [25].

Katz et al. aimed to augment the host T-cell response by delivering regional hepatic CAR-T cells targeting carcinoembryonic antigen (CEA) [17]. The high levels of CEA in colorectal cancer and its status as a quantifiable biomarker make it an appealing CAR target [17]. While previous studies had examined systemic delivery of CEA targeting CAR-T cells, these trials were limited by severe colitis [10]. The investigators conducted this phase I study testing the safety and efficacy of hepatic artery infusions (HAI) of anti-CEA CAR-T cells with and without IL-2 co-administration in six patients with unresectable, CEA positive, solitary hepatic metastases.

**Figure 2:**
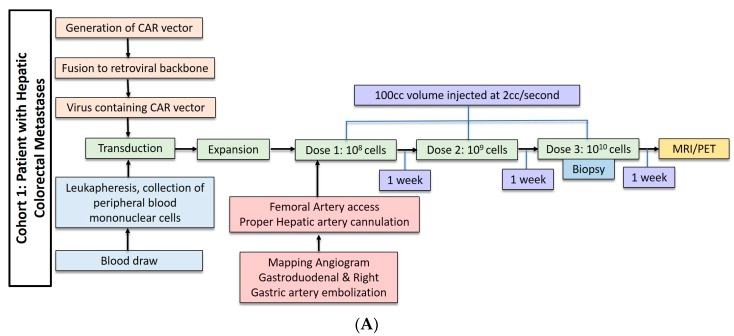

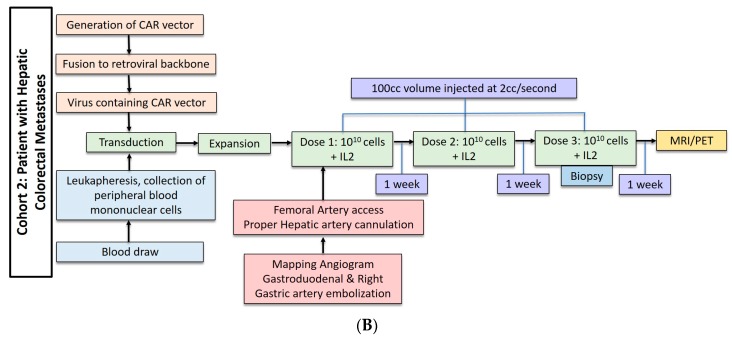
(**A**) Dose regimen for hepatic arterial infusion of CAR-T cells without IL2. (**B**) Dose regimen for hepatic arterial infusion of CAR-T cells with IL2 co-treatment.

Three patients underwent anti-CEA CAR-T HAI without IL-2 administration. These patients received dose escalation from 10^8^ cells to 10^10^ cells (Figure 2A). The second cohort of three patients received three doses of 10^10^ cells with 75,000 U/kg/day of systemic IL-2 (Figure 2B). The CAR-T cells were administered during hepatic angiogram approached via the common femoral artery. To limit extrahepatic administration, the right gastric and the common hepatic arteries were coil embolized. The proper hepatic artery was then cannulated and CAR-T cells were infused through a 60 cc syringe by hand injection at a rate of less than 2 cc per second. A total volume of 100 cc was injected. Contrast angiography performed halfway through injection and at completion confirmed intact hepatic arterial inflow. Of note, in cases of aberrant hepatic artery anatomy, angiography was used to identify the right and left hepatic artery and half doses were administered to each.

Following CAR-T infusions, safety data, cell trafficking data, and clinical activity were measured. There were no life threatening or fatal adverse events (Common Terminology Criteria for Adverse Events 3.0) in this study. In the cohort of patients who did not receive IL-2, all patients tolerated CAR-T infusions of 10^10^ cells without the need for dose reduction. When this dose was co-administered with IL-2, the most common adverse event was febrile episodes. In one patient, fevers, tachycardia, and reversible eosinophilia prompted a 50% dose reduction. In all patients in the IL-2 cohort, interruption of IL-2 administration was required secondary to grade 1–3 adverse events. Biopsies of normal liver parenchyma following infusion revealed that there were no histological adverse effects despite transient transaminitis in all patients. Trafficking of CAR-T cells was measured by flow cytometry taken from biopsies of liver metastases, normal liver parenchyma, and peripheral blood samples before the start of therapy and at the time of the last CAR-T dose. Notably, CAR-T cells were found within biopsies of liver metastases in five of six patients, with one patient showing evidence of intratumoral CAR-T cell persistence at three months following the final infusion. Serum CAR-T cells were detected during infusion in two patients, however no measurable CAR-T was detected in peripheral blood samples as early as three days following the last infusion, and as late as one month following this infusion.

Clinical activity specific to CAR-T therapy was assessed by measuring serum CEA levels at serial time points following infusions for each patient. In the non-IL-2 cohort, two of three patients experienced transient decreases in serum CEA levels, while the one patient who did not showed the shortest survival following therapy. Patients in the IL-2 cohort had a more robust response to therapy as measured by serum CEA concentration. Serum CEA in all three patients in this cohort decreased, with one patient’s serum CEA dropping by 48% percent. One patient from this group experienced a survival of 102 weeks following infusion and subsequently received microwave ablation of unresectable disease. Histological review of biopsies of liver metastases showed increased necrosis in three patients, and intratumoral necrosis and fibrosis in four patients.

## 3. Discussion and Conclusions

Regional delivery of CAR-T cells appears to be safe and feasible with promising clinical efficacy in patients with glioblastoma and colorectal cancer with liver metastases. Regional delivery leads to effective CAR-T cell trafficking and avoids the physical barrier of the tumor microenvironment relative to systemic circulation; however, several limitations still exist regarding its use. None of the clinical trials examining regional delivery of CAR-T cells resulted in fatal or life-threatening toxicities, but fever and tachycardia were common adverse events and may represent a systemic inflammatory response despite localized delivery of therapy. Notably, upon hepatic artery infusion of anti-CEA CAR-T cells by Katz et al. CAR-T cells were transiently present in circulation immediately following infusion, theoretically placing this patient at risk of toxicity associated with systemic delivery. No toxicity was seen in this patient, but the nature of toxicity associated with intravenous versus local delivery of these cells should be elucidated in future studies. Katz et al. displayed evidence of intralesional CAR-T 12 weeks from the end of therapy in colorectal liver metastases [17]. While intralesional CAR-T cells were present five weeks after the start of therapy in one trial from Brown et al., it should be noted that therapy was delivered over a five-week period [12]. The time range of intralesional persistence of CAR-T cells remains unknown after both studies. Remarkable clinical efficacy was seen in one patient with metastatic glioblastoma for 7.5 months during active intracavitary and intratumoral delivery of CAR-T cells. Intriguingly, it was observed that the accumulation of T-cells in the CSF was limited. Rather, there was an increase of inflammatory cytokines of the CSF [13]. The mechanism of the multifocal response in this patient is not yet clear, and warrants further investigation. In the examined clinical trials, antigen levels have been used as a tool to measure response to CAR-T therapy. Despite decreased levels of the targeted antigens, all the patients in these studies except one experienced recurrence or disease progression. Whether this occurs because of antigen depletion or via a different mechanism of resistance should also be the subject of ongoing research. The addition of regional CAR-T therapy to other modalities of care such as chemotherapy and radiation may provide a method to avoid or overcome resistance, and is being examined currently in a phase I study examining hepatic intratumoral CAR-T therapy with concurrent administration of radiation emitting microspheres for hepatic metastases (NCT02416466) [26].

In addition to the results from the clinical trials discussed, Table 1 presents recent pre-clinical studies that have demonstrated potential safety benefits of local delivery of CAR-T cells to the pleural cavity and the peritoneal cavity for the treatment of mesothelioma and peritoneal metastases, respectively [11,18,27]. Most interesting in these pre-clinical studies was the difference in T-cell persistence demonstrated after regional delivery as opposed to systemic delivery. Adusumilli et al. revealed that intrapleural delivery of CAR-T cells not only demonstrated trafficking ability, thereby inhibiting tumor growth at distant locations, but persistence as effector T-cells for up to 100 days following delivery [11]. In contrast, van der Stegen et al. were not able to detect CAR-T cell persistence past five days [28]. Pleural delivery of CAR-T cells is now being explored in a phase I clinical trial building off these preclinical studies (NCT02414269) [29]. Given the lack of consistent pre-clinical evidence, an important secondary outcome of these clinical trials will be the persistence of CAR-T cells.

The use of regional CAR-T delivery as an adjunct to surgical therapy for control of locally advanced disease is novel and exciting. Head and neck cancer often presents in locally advanced stages that may preclude curative resections and increase the likelihood of recurrence. Aside from surgery, locoregional therapy is only provided with radiation therapy. The high rate of recurrence and mortality associated with advanced locoregional disease presents an opportunity for the advent of newer and less morbid therapies. Pre-clinical studies have investigated the in vivo activity of the nonspecific ErbB ligand binding CAR, T1E28z, against intraperitoneally implanted head and neck cell line derived xenografts [30]. Intraperitoneal delivery of CAR-T cells in this study was efficacious, and subsequent studies by van der Stegen et al. showed that cytokine release syndrome was associated with intravenous delivery compared to intraperitoneal delivery [28]. On the basis of these results, a phase I clinical trial utilizing intratumoral delivery of T1E28z CAR-T cells in head and neck cancer is currently underway (NCT01818323) [31]. Intratumoral delivery of ErbB2-targeted CAR-T cells has also been examined in pre-clinical studies of ErbB2 over-expressing transgenic mice, and will likely be subject to further studies given the status of this ligand in multiple types of adenocarcinoma, including head and neck, lung, breast, prostatic, pancreatic, colon, and gastric [32].

Despite some caveats, CAR-T therapy remains an exciting field with promise, and regional delivery of CAR-T cells may provide another arrow in the quiver of clinical oncologists.

## Figures and Tables

**Table 1:** Summary of clinical and select pre-clinical studies of regional delivery of CAR-T cells.

Author	Cancer	Study Type	CAR	Stimulatory Signal	Inhibitory Signal	Additional Therapy	Delivery Method
Yaghoubi et al. [19]	Glioblastoma	Clinical Phase I	IL13Rα2	-	-	Surgical Resection	Catheter infusion into resection cavity
Brown et al. [12]	Glioblastoma	Clinical Phase I	IL13Rα2	-	-	Surgical Resection	Catheter infusion into resection cavity
Brown et al. [13]	Glioblastoma	Clinical Phase I	IL13Rα2	CD137	IgG4-Fc mutant ^A^	Surgical Resection	Catheter infusion into resection cavity
Katz et al. [17]	Hepatic colorectal metastases	Clinical Phase I	CEA	IL2	-	Chemotherapy	Hepatic artery infusion
Katz et al. [26]	Hepatic colorectal metastases	Clinical Phase I	CEA	IL2	-	Chemotherapy + Yttrium spheres	Hepatic Artery Infusion
van Schalkwyk et al. [31]	Head and Neck	Clinical Phase I	T1E28z (ErbB) ^B^	Cytokine receptor 4αβ ^C^	-	Cyclophoshamide	Ultrasound guided intratumoral injection
Adusumilli et al. [29]	Pleural Mesothelioma	Clinical Phase I	M28z ^D^	CD28	-	-	Intrapleural
Katz et al. [18]	Peritoneal carcinomatosis	Pre-clinical	CEA	IL2	Anti-PDL1 Anti-Gr1 Anti-GITR ^E^	-	Intraperitoneal
Adusumilli et al. [11]	Pleural Mesothelioma	Pre-clinical	M28z	CD28	-	Cyclophosphamide	Intrapleural
van der Stegen et al. [28]	Head and Neck	Pre-clinical	T1E28z (ErbB)	Cytokine receptor 4αβ ^C^	-	-	Intraperitoneal
Davies et al. [30]	Head and Neck	Pre-clinical	T1E28z (ErbB)	Cytokine receptor 4αβ ^F^	-	-	Intraperitoneal
Klampatsa et al. [27]	Mesothelioma	Pre-clinical	T1E28z (ErbB)	Cytokine receptor 4αβ	-	-	Intraperitoneal

^A^ Fc region mutation to reduce the off-target Fc receptor interactions. ^B^ A chimeric antigen formed by transforming growth factor-α and epidermal growth factor that recognizes several ErbB dimers. ^C^ Chimeric cytokine receptor between IL-4 receptor α ectodomain and IL-2/IL-15 β chain allowing for ex vivo growth stimulation with IL-4 treatment. ^D^ Mesothelin specific single-chain variable fragment with CD28/CD3ζ signaling domains. ^E^ Anti-Programmed dealth-ligand 1, Anti-Granulocyte Receptor 1, Anti-Glucocorticoid induced tumor necrosis factor receptor 1. ^F^ IL-4 administered in vivo rather than ex vivo.

## References

[1] Newick K., Brien S.O., Moon E., Albelda S.M. (2017). CAR-T cell therapy for solid tumors. Annu. Rev. Med..

[2] Newick K., Moon E., Albelda S.M. (2016). Chimeric antigen receptor T-cell therapy for solid tumors. Oncolytics.

[3] Zhang E., Xu H. (2017). A new insight in chimeric antigen receptor-engineered T cells for cancer immunotherapy. J. Hematol. Oncol..

[4] Geldres C., Savoldo B., Hoyos V., Caruana I., Zhang M., Yvon E., Vecchio M.D., Creighton C.J., Ittmann M.T. (2015). T Lymphocytes Redirected against the Chondroitin Sulfate Proteoglycan-4 Control the Growth of Multiple Solid Tumors both In Vitro and In Vivo. Clin. Cancer Res..

[5] Beard R.E., Zheng Z., Lagisetty K.H., Burns W.R., Tran E., Hewitt S.M., Abate-daga D., Rosati S.F., Fine H.A., Ferrone S. (2014). Multiple chimeric antigen receptors successfully target chondroitin sulfate proteoglycan 4 in several different cancer histologies and cancer stem cells. J. Immunother. Cancer.

[6] Posey A.D., Schwab R.D., Boesteanu A.C., Steentoft C., Mandel U., Engels B., Stone J.D., Madsen T.D., Schreiber K., Haines K.M. (2016). Engineered CAR T Cells Targeting the Cancer- Associated Tn-Glycoform of the Membrane Mucin MUC1 Control Adenocarcinoma. Immunity.

[7] Prapa M., Caldrer S., Spano C., Bestagno M., Golinelli G., Grisendi G., Petrachi T., Conte P., Horwitz E.M., Paolucci P. (2015). A novel anti-GD2/4–1BB chimeric antigen receptor triggers neuroblastoma cell killing. Oncotarget.

[8] Li H., Zhao Y. (2017). Increasing the safety and ef fi cacy of chimeric antigen receptor T cell therapy. Protein Cell.

[9] Maus M.V., June C.H. (2016). CCR FOCUS Making Better Chimeric Antigen Receptors for Adoptive T-cell Therapy. Clin. Cancer Res..

[10] Parkhurst M.R., Yang J.C., Langan R.C., Dudley M.E., Nathan D.-A.N., Feldman S.A., Davis J.L., Morgan R.A., Merino M.J., Sherry R.M. (2011). T cells targeting carcinoembryonic antigen can mediate regression of metastatic colorectal cancer but induce severe transient colitis. Mol. Ther..

[11] Adusumilli P.S., Cherkassky L., Villena-vargas J., Servais E., Plotkin J., Jones D.R., Sadelain M. (2015). Regional delivery of mesothelin-targeted CAR T cell therapy generates potent and long-lasting CD4-dependent tumor immunity. Sci. Transl. Med..

[12] Brown C.E., Badie B., Barish M.E., Weng L., Julie R., Chang W., Naranjo A., Starr R., Wagner J., Wright C. (2016). Bioactivity and safety of IL13Ra2-redirected chimeric antigen receptor CD8+ T cells in patients with recurrent glioblastoma. Clin. Cancer Res..

[13] Brown C.E., Alizadeh D., Ostberg J.R., Blanchard M.S., Kilpatrick J., Simpson J., Kurien A., Priceman S.J., Wang X. (2016). Regression of glioblastoma after chimeric antigen receptor T-cell therapy. N. Engl. J. Med..

[14] Choi D.B., Suryadevara M.C., Gedeon C.P., Herndon E.J., Sanchez-Perez L., Sampson H.J. (2015). Intracerebral delivery of a third generation EGFRvIII-specific chimeric antigen receptor is efficacious against human glioma. J. Clin. Neurosci..

[15] Han J., Chu J., Chan W.K., Zhang J., Wang Y., Cohen J.B., Victor A., Meisen W.H., Kim S., Grandi P. (2015). CAR-engineered NK cells targeting wild-type EGFR and EGFRvIII enhance killing of glioblastoma and patient-derived glioblastoma stem cells. Sci. Rep..

[16] Kahlon K.S., Brown C., Cooper L.J.N., Raubitschek A., Forman S.J., Jensen M.C. (2004). Specific recognition and killing of glioblastoma multiforme by interleukin 13-zetakine redirected cytolytic T cells. Cancer Res..

[17] Katz S.C., Burga R.A., Mccormack E., Wang L.J., Mooring W., Point G., Khare P.D., Thorn M., Ma Q., Stainken B.F. (2015). Phase I hepatic immunotherapy for metastases study of intra-arterial chimeric antigen receptor modified T cell therapy for CEA^+^ liver metastases. Clin. Cancer Res..

[18] Katz S., Point R.G., Cunetta M., Thorn M., Guha P., Espat N.J., Boutros C., Hanna N., Junghans P.R. (2016). Regional CAR-T cell infusions for peritoneal carcinomatosis are superior to systemic delivery. Cancer Gene Ther..

[19] Yaghoubi S.S., Jensen M.C., Satyamurthy N., Paik D., Czernin J., Gambh S.S. (2009). Non-invasive detection of therapeutic cytolytic T Cells in patients with [18-F]FHBG positron emission tomography in a glioma patient. Nat. Clin. Pract. Oncol..

[20] Paty P., Kemeny N., Tong W., Sullivan D., Fong Y., Jarnagin W. (2000). A phase I/II study of hepatic arterial infusion (HAI) of floxuridine (FUDR) and dexamethasone (DEX) with systemic irinotecan (CPT-11) for unresectable hepatic metastases from colorectal cancer. Proc. Am. Soc. Clin. Oncol..

[21] Kemeny N., Jarnagin W., Paty P., Gönen M., Schwartz L., Morse M., Leonard G., D’Angelica M., DeMatteo R., Blumgart L. (2005). Phase I trial of systemic oxaliplatin combination chemotherapy with hepatic arterial infusion in patients with unresectable liver metastases from colorectal cancer. J. Clin. Oncol..

[22] Kemeny N.E., Huitzil Melendez F.D., Capanu M., Paty P.B., Fong Y., Schwartz L.H., Jarnagin W.R., Patel D., D’Angelica M. (2009). Conversion to resectability using hepatic artery infusion plus systemic chemotherapy for the treatment of unresectable liver metastases from colorectal carcinoma. J. Clin. Oncol..

[23] Corrigan-Curay J., Kiem H.-P., Baltimore D., O’Reilly M., Brentjens R.J., Cooper L., Forman S., Gottschalk S., Greenberg P., Junghans R. (2014). T-Cell immunotherapy: Looking forward. Mol. Ther..

[24] Misiakos E.P., Nikolaos P., Kouraklis G. (2011). Current treatment for colorectal liver metastases. World J. Gastroenterol..

[25] Guha P., Reha J., Katz S.C. (2016). Immunosuppression in liver tumors: Opening the portal to effective immunotherapy. Nat. Publ. Gr..

[26] U.S. National Institutes of Health Phase Ib Trial of CAR-T Hepatic Artery Infusions Followed by Selective Internal Radiation Therapy (SIRT) with Yttrium-90 Sir-Spheres® for CEA-Expressing Liver Metastases. NCT02414269.

[27] Klampatsa A., Achkova D.Y., Davies D.M., Parente-Pereira A.C., Woodman N., Rosekilly J., Osborne G., Thayaparan T., Bille A., Sheaf M. (2017). Intracavitary “T4 immunotherapy” of malignant mesothelioma using pan-ErbB re-targeted CAR T-cells. Cancer Lett..

[28] Van der Stegen S.J.C., Davies D.M., Wilkie S., Foster J., Sosabowski J.K., Burnet J., Whilding L.M., Petrovic R.M., Ghaem-Maghami S., Mather S. (2013). Preclinical in vivo modeling of cytokine release syndrome induced by ErbB-retargeted human T cells: Identifying a window of therapeutic opportunity?. J. Immunol..

[29] U.S. National Institutes of Health Malignant pleural disease treated with autologous T-cells genetically engineered to target the cancer-cell surface antigen mesothelin. NCT02414269.

[30] Davies D., Foster J. (2012). Flexible Targeting of ErbB dimers that drive tumorigenesis by using genetically engineered T cells. Mol. Med..

[31] Van Schalkwyk M.C.I., Papa S.E., Jeannon J.-P., Guerrero Urbano T., Spicer J.F., Maher J. (2013). Design of a phase I clinical trial to evaluate intratumoral delivery of ErbB-targeted chimeric antigen receptor T-cells in locally advanced or recurrent head and neck cancer. Hum. Gene Ther. Clin. Dev..

[32] Globerson-Levin A., Waks T., Eshhar Z. (2014). Elimination of progressive mammary cancer by repeated administrations of chimeric antigen receptor-modified T cells. Mol. Ther..

